# Development of an Adaptable Qualification Test Set for Personnel Involved in Visual Inspection Procedures of Parenteral Drug Products Manufactured Under Good Manufacturing Practice Conditions in Hospital Pharmacy Compounding Facilities

**DOI:** 10.3390/pharmaceutics17010074

**Published:** 2025-01-07

**Authors:** Tessa van den Born-Bondt, Harmen P. S. Huizinga, Koen R. Kappert, Hans H. Westra, Jacoba van Zanten, Herman J. Woerdenbag, Jacoba M. Maurer, Bahez Gareb

**Affiliations:** 1 Department of Clinical Pharmacy and Pharmacology, University Medical Center Groningen (UMCG), 9713 GZ Groningen, The Netherlands; 2 Department of Pharmaceutical Technology and Biopharmacy, Groningen Research Institute of Pharmacy (GRIP), University of Groningen, Antonius Deusinglaan 1, 9713 AV Groningen, The Netherlands

**Keywords:** good manufacturing practice (GMP), qualification, visual inspection, parenteral drugs, hospital pharmacy, pharmaceutical compounding, qualification test set, particulate matter

## Abstract

**Objectives:** Parenteral drug products manufactured under GMP conditions should be visually inspected for defects and particulate contamination by trained and qualified personnel. Although personnel qualification is required, no practical protocols or formal guidelines are available for the development of qualification test sets (QTSs) used for qualification procedures. The current practice is to either procure a standardized QTS from a commercial supplier or amass sufficient manufacturing rejects during visual inspection procedures to compile in-house QTSs. However, both strategies inherently possess disadvantages and limitations. The objective of this study was to develop a manufacturing protocol for an optimal and adaptable QTS for training and qualification procedures. **Methods:** We combined the results of a literature search, survey of five Dutch hospital pharmacy compounding facilities, semi-structured personnel interviews, and extensive pre-GMP formulation studies to develop an optimal and adaptable QTS manufacturing protocol. **Results:** The literature search did not identify a manufacturing protocol for an optimal and adaptable QTS, but did identify specifications and requirements for optimal QTSs. The survey among hospital pharmacy compounding facilities revealed considerable variability in the qualification procedures and used QTSs. Semi-structured personnel interviews and pre-GMP formulation studies demonstrated that defects encountered during routine productions could be realistically simulated with pharmaceutical-grade excipients. As a proof-of-concept, we manufactured two different QTSs under GMP conditions and assessed these for formal GMP training and qualification purposes, which were considered a significant improvement compared to using manufacturing rejects. **Conclusions:** To the best of our knowledge, this is the first study presenting these data and our adaptable protocol, which is provided in the Supplemental Materials, may aid compounding facilities in the standardization, training, and qualification of personnel involved in visual inspection procedures.

## 1. Introduction

Hospital pharmacy compounding facilities play an essential role in providing patients with extemporaneous preparations when no adequate pharmaceutical alternatives are commercially available. Moreover, hospitals with a GMP manufacturing license also play an essential role in providing clinicians and investigators with experimental medication intended for clinical trials (phase I/II). Small-scale productions, such as those for individual patients or clinical trial medication at limited costs, are often not facilitated by pharmaceutical companies. Additionally, hospital pharmacy compounding facilities usually have a wider range of products compared to a pharmaceutical company, including aseptically prepared, terminally sterilized, and non-sterile preparations. Taken together, these activities highlight the indispensable contribution of hospital pharmacies to innovation and personalized treatment of patients.

Parenteral preparations are sterile drug products that are either aseptically manufactured or terminally sterilized. All parenteral drug products manufactured under GMP conditions should be visually inspected by qualified personnel for possible particulate contamination [1]. Particulate contamination is defined as the presence of extrinsic and/or intrinsic particulate matter in the drug product [2]. Extrinsic particles are not part of the formulation, packaging, or assembly process. They include, for example, clothing fragments, fibers, and hair. Intrinsic particles are particles that can be attributed to the formulation, packaging, and assembly process, such as glass and rubber particles originating from the primary packaging material or precipitates due to instable active pharmaceutical ingredients or excipients [3]. Since the source and characteristics of these particles can vary, so do their size and visibility under standardized lighting conditions.

There is no single particle size defined as the smallest observable size by the human eye. The United States Pharmacopeia (USP) <1787> monograph considers particles larger than 100 μm to be visible [4]. However, under conditions of increased light intensity, particles smaller than 100 μm may be distinguished by the human eye [5].

The European Pharmacopoeia (Ph. Eur.) method 2.9.20 and USP monograph <1790> describe the visual inspection methods and acceptance criteria for parenteral drugs manufactured in the European Union (EU) and United States of America (USA), respectively [6,7]. In general, these methods require that the drug products are visually inspected in bright light against white and black backgrounds. Any observed defect to the primary container or the presence of particulate matter in the drug product should be rejected and discarded [8].

The EU GMP Annex 1 states that all manufacturing activities should be performed by trained and qualified personnel [1]. Guidelines describing the qualification of personnel involved in visual inspection procedures of parenteral drugs state that inspectors must demonstrate proficiency of removing defects from a seeded population of typical “in-house” defects. Before being trained and formally qualified, an operator should undergo a visual acuity test [1]. Subsequently, personnel are qualified using qualification test sets (QTSs) containing representative defects that are typically encountered during routine productions. To warrant the qualification status, personnel should be requalified with a predetermined and justified frequency, which is typically at least annually [8].

An optimal QTS should meet a number of requirements [8,9,10]. First, it should contain a subset of defect-free containers and a subset (10–20% of the total) of containers containing at least one defect. This ensures that personnel are not only qualified to detect the presence of any defects but also qualified to accept containers without defects. Second, the QTS should contain a representative number of units (batch size) to simulate a typical visual inspection procedure encountered during routine productions. Furthermore, the QTS units should ideally be identical to units manufactured during routine productions, i.e., similar primary packaging materials, fill volumes, and color/turbidity of solution. Third, the QTS should contain a wide range of representative defects (e.g., particles, fibers, and damages) to optimally qualify the personnel for the defects that can be encountered during routine productions. Finally, in view of practicality, the QTS should have a long shelf life (years) and should withstand repeated handling over time [9].

The units of the QTS may be sourced from actual manufacturing rejects encountered during routine productions. It is also possible to procure standardized QTSs from commercial suppliers [8]. However, both strategies inherently possess disadvantages. For instance, using manufacturing rejects may not yield a wide range of defects in a timely manner, especially if the compounding facility manufactures multiple drug product formulations (e.g., syringes, bags for intravenous infusion, containers, and ampoules) with different fill volumes in relatively small batch sizes (e.g., 100–200 units). Furthermore, commercially available QTSs are expensive, especially when multiple sets need to be procured to train personnel for qualification procedures of multiple drug product formulations. Moreover, such sets may lack representative units or defects typically encountered during routine productions. The high costs associated with commercially available QTSs are generally the result of the standardized particle size and count present in the containers. However, these features are not necessary nor required for the qualification of personnel since the qualification procedure is a qualitative (present/absent of defect) rather than a quantitative test (not tested for particle size and count).

As an alternative, QTSs may be manufactured by the compounding facility itself. This strategy is relatively easy, cheap, and adaptable and yields fully representative QTS units with the possibility to include colored, viscous, and opalescent solutions (if desired) in the same packaging materials as used during routine productions. Furthermore, the batch size of the manufactured QTS can be as large as needed, which ensures that the QTS simulates the circumstances of a visual inspection procedure during routine productions. In addition, each desired and representative defect can be introduced in any given frequency based on historic visual inspection or trend analysis data. These in-house manufactured QTSs are thus more representative for routine practice and provide a more reliable qualification procedure. Although such QTSs are more suitable for training and qualification procedures, methods and requirements to manufacture these test sets are lacking in the current literature.

The objective of this study was to develop a manufacturing protocol for an adaptable QTS that can be used for the qualification of personnel involved in visual inspection procedures of parenteral drug products manufactured under GMP conditions. First, a literature search was performed to investigate whether manufacturing protocols and requirements for a QTS are available. Second, a survey was conducted to assess the visual inspection qualification procedures and used QTSs in five Dutch hospital compounding pharmacies. Subsequently, personnel involved in visual inspection procedures were interviewed to gather data on typical and rarer defects encountered during routine visual inspection procedures and these defects characteristics. Based on these results, QTS requirements and formulation studies were conducted to develop a manufacturing protocol for an adaptable and in-house manufactured QTS ready for GMP tech transfer. As a proof-of-concept, two QTSs were manufactured under GMP conditions with the developed manufacturing protocol and were evaluated for feasibility and suitability for visual inspection qualification procedures under GMP conditions.

## 2. Materials and Methods

### 2.1. Study Design

This study was conducted in the compounding facility of the University Medical Center Groningen (UMCG), The Netherlands, which is a tertiary hospital with a GMP manufacturing license for parenteral drug products manufactured under EU GMP Annex 1 conditions. A wide range of parenteral drug products are manufactured in our compounding facility, including fluorescent-labelled monoclonal antibodies.

**Figure 1:**
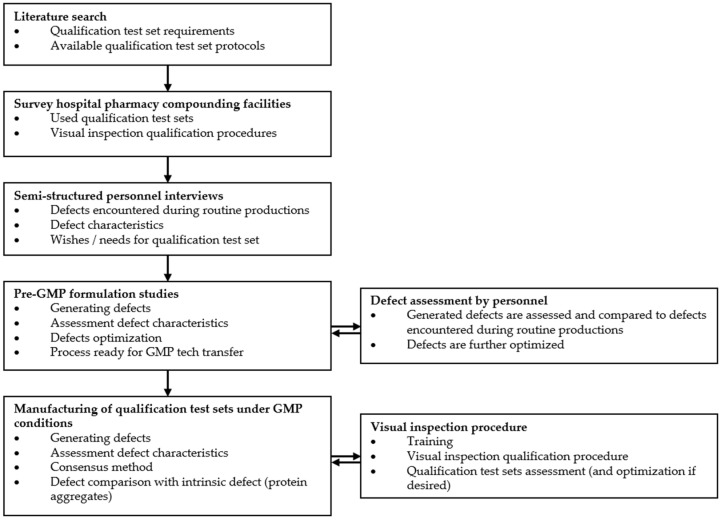
Study design and flow chart of the present study.

We used a mixed methods study design, combining the results of a literature search, survey of five Dutch hospital pharmacy compounding facilities (including our own facility), semi-structured interviews of qualified personnel involved visual inspection procedures of parenteral drugs, and pre-GMP formulation studies to manufacture two QTSs under GMP conditions with the developed manufacturing protocol (Figure 1).

To show proof-of-concept, the present study focused on fluorescent-labeled monoclonal antibodies, in particular, bevacizumab-800CW [11]. This parenteral drug product is a clear, blue-greenish, aqueous solution with a protein concentration of 1 mg/mL and packaged in clear type I glass 10R vials (APG Europe, Uithoorn, The Netherlands) with a bromobutyl rubber stopper (Brocacef Supplies & Services, Maarssen, The Netherlands) and aluminum crimp with a plastic cap (Brocacef Supplies & Services, Maarssen, The Netherlands) with a fill volume of 5–6 mL and with a batch size of 100–200 units. Possible causes and sources of particulate contamination or defective containers were identified with a risk assessment using a Ishikawa diagram (Appendix A).

### 2.2. Literature Search

To investigate whether manufacturing protocols and requirements for a QTS are available in the literature, the PubMed, Embase, and Web of Science databases were searched.

The search term for the PubMed database was ‘(qualification[tiab] OR qualification set[tiab] OR test set[tiab] OR training set[tiab] OR challenge set[tiab]) AND (good manufacturing practices[tiab] OR GMP[tiab] OR visual inspection[tiab] OR parenteral[tiab] OR visible particles[tiab] OR particles[tiab] OR particulate matter[tiab])’.

The search term for the Embase database was ‘(‘qualification’:ti,ab OR ‘qualification set’:ti,ab OR ‘test set’:ti,ab OR ‘training set’:ti,ab OR ‘challenge set’:ti,ab) AND (‘good manufacturing practice’:ti,ab OR ‘GMP’:ti,ab OR ‘visual inspection’:ti,ab OR ‘parenteral’:ti,ab OR ‘visible particles’:ti,ab OR ‘particles’:ti,ab OR ‘particulate matter’:ti,ab)’.

The search term for the Web of Science database was ‘(TI=(qualification) OR AB=(qualification) OR TI=(qualification set) OR AB=(qualification set) OR TI=(test set) OR AB=(test set) OR TI=(training set) OR AB=(training set) OR TI=(challenge set) OR AB=(challenge set)) AND (TI=(good manufacturing practices) OR AB=(good manufacturing practices) OR TI=(GMP) OR AB=(GMP) OR TI=(visual inspection) OR AB=(visual inspection) OR TI=(parenteral) OR AB=(parenteral) OR TI=(visible particles) OR AB=(visible particles) OR TI=(particulate matter) OR AB=(particulate matter))’.

The titles as well as abstracts of all articles were read to assess whether the articles described manufacturing protocols or requirements for QTSs. The reference list of articles describing QTS manufacturing protocols or requirements were also screened for potential relevant articles.

To investigate the requirements for a QTS intended for GMP qualification purposes, guidelines from the EU GMP (Annex 1), FDA, Parental Drug Association (PDA), USP, British Pharmacopoeia (BP), and Ph. Eur. were reviewed and summarized [1,6,7,8,10,12]. The obtained data were used for the development of the manufacturing protocol described in the present study and to warrant that the QTS complied with the relevant guidelines for manufacturing parenteral drug products.

### 2.3. Survey Hospital Pharmacy Compounding Facilities

To assess the current practices in GMP qualification of personnel involved in the visual inspection of parental drug products, a survey was distributed to six different hospital pharmacy compounding facilities in the Netherlands with a GMP manufacturing license to manufacture parenteral drug products (including the UMCG compounding facility). The survey was conducted via e-mail and aimed to assess the general qualification procedure, the used QTSs, and coding/decoding method of the test set. The survey questions of this survey are presented in Appendix B.

### 2.4. Semi-Structured Personnel Interviews

Semi-structured interviews were conducted with qualified personnel (four participants) involved in visual inspection procedures of parenteral drug products manufactured under GMP conditions by the UMCG. The interview questions were directed to identify which routine defects were typically encountered with what frequency, what typical defect characteristics (e.g., size, motion, ease of dispersion, sedimentation rate, and interaction with light in solution) were during visual inspection procedures according to Ph. Eur. 2.9.20, which defects were easy/difficult to spot, and what the specific wishes of the personnel were regarding the training and qualification procedures. Open-ended questions were used with additional probing questions when necessary to avoid researcher bias. The semi-structured interview questions are available in Appendix C.

### 2.5. Pre-GMP Formulation Studies

Pre-GMP formulation studies were conducted to assess the suitability of different pharmaceutical excipients and cleanroom materials for the QTS defects that were identified in the semi-structured interviews (see Section 2.4). Pre-GMP production was performed in a laminar airflow cabinet (LAF) with EU GMP grade A air situated in an unclassified area.

Ideally, excipients compatible with GMP conditions should be used, since the final production process must comply with GMP standards. Therefore, Ph. Eur. and GMP-grade materials, such as pharmaceutical excipients and sterile wipes, were chosen. Talc (VWR, Radnor, PA, USA), microcrystalline cellulose ((MCC), Pharmacel^®^ 102, particle size distribution ×10: 40 µm; ×50: 90 µm; ×90: 180 µm, DFE Pharma, Goch, Germany), barium sulfate (Thermo Scientific, Waltham, MA, USA), magnesium stearate (Spruyt Hillen, Capelle aan den IJssel, The Netherlands), sodium carbonate (Merck, Darmstadt, Germany), and silicon dioxide (colloidal anhydrous silica) (Merck, Darmstadt, Germany) were selected to simulate particulate matter.

Talc particles were generated either from talc agglomerates directly deposited into the glass vials or by first depositing talc agglomerates onto the surface of water for injection (WFI), which remained afloat by the surface tension of the water. Subsequently, a small portion of the agglomerate was transferred into the sterile 10R vials (Ph. Eur. Type I glass, APG Europe, Uithoorn, The Netherlands). MCC particles were generated by first transferring a small amount of MCC into a glass beaker filled with WFI. A small portion of MMC particles was subsequently transferred into the glass vial with a spatula.

Glass shards were obtained by gently chipping the tip of a 150 mm Pasteur capillary pipette (Thermo Scientific, Waltham, MA, USA) with sterile tweezers. Rubber particles were generated by cutting a bromobutyl rubber stopper (Brocacef Supplies & Services, Maarssen, The Netherlands), which was part of the primary packaging, with a sterile surgical scalpel. Fibers were produced using either a paper towel (Tork, Zeist, The Netherlands) or a Sontara Micropure AP sterile cloth (IPS-Group A/S, Kvistgård, Denmark). Suitable fibers were then transferred into the containers containing WFI using sterile tweezers.

**Figure 2:**
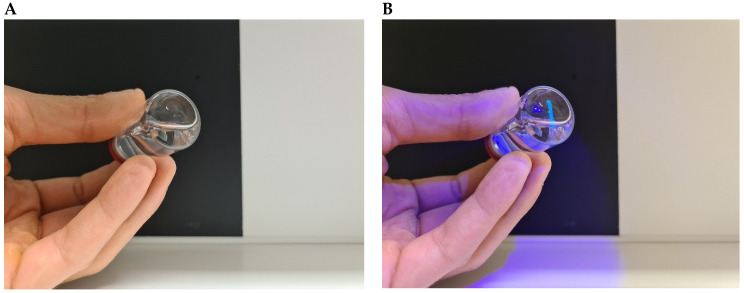
Coding and decoding of the QTS vials with a UV marker and UV lamp. (**A**) The test set vial code cannot be seen in visible light during visual inspection according to Ph. Eur. 2.9.20. (**B**) The code (“number 1”) can be made visible with a UV lamp, which warrants a fully blinded qualification procedure of personnel.

After the defects consisting of particulate matter were generated, the glass vials were filled with 5–6 mL WFI and sealed with a bromobutyl rubber stopper and aluminum crimp with a plastic cap. Furthermore, dents and chips were generated as defects in the aluminum crimp or plastic cap, respectively, by mechanical force applied to vials that did not contain particulate matter as a defect. All vials were coded with a number using a UV marker (Edding 8280, Ahrensburg, Germany). The mark could be made visible with a UV lamp (BASEY, BH-ZaklampUV, Nieuw Vennep, The Netherlands) (Figure 2).

The manufactured defects were assessed by qualified UMCG personnel involved in visual inspection procedures. The appearance as well as flow characteristics of particulate matter during visual inspection according to Ph. Eur. 2.9.20 (Apollo II Liquid Viewer, Aldephi, United Kingdom) was assessed. In case the generated defects did not accurately simulate the defects encountered during routine GMP productions, defects were further formulated and optimized. This process was repeated until the generated defects accurately simulated the defects encountered during routine GMP productions and therefore was ready for tech transfer (Figure 1).

### 2.6. Manufacturing of Qualification Test Sets Under GMP Conditions

Two final GMP QTSs were manufactured according to a summary of the guidelines described in Section 2.2 and the procedures described in Section 2.5. The only difference between the pre-GMP and GMP QTSs was that the two GMP QTSs were manufactured under GMP conditions in a LAF cabinet with EU GMP-grade A air situated in an EU GMP-grade C cleanroom with a batch size of 100 units.

The first GMP QTS consisted of the primary container filled with 5–6 mL WFI with and without defects. The second GMP QTS consisted of the primary container filled with 5–6 mL WFI in which the fluorescent dye IRDye 800CW (LI-COR, Lincoln, NE, USA) was dissolved at a concentration of 10 µg/mL to mimic the color of the fluorescent-labelled monoclonal antibodies (e.g., bevacizumab-800CW [9]) that are routinely manufactured as clinical trial medication by the UMCG.

After the manufacturing process and batch release, the GMP QTSs were simultaneously visually inspected by two qualified persons according to Ph. Eur. 2.9.20 (Apollo II Liquid Viewer, Aldephi, UK). The objective of this inspection procedure was to confirm with the four-eye principle (i.e., consensus method) that the vials containing the defects were identified as such and to confirm that the vials that did not contain any defects were indeed identified as such.

### 2.7. Comparison Qualification Test Set Defects with Protein (Monoclonal Antibody) Aggregates

To assess whether the generated defects consisting of particulate matter simulated protein aggregates (intrinsic particles), GMP-produced bevacizumab-800CW vials were stored at 50 °C for 48 h in an oven (Salm & Kipp, Breukelen, The Netherlands). Subsequently, these vials were stored for an additional 7 days at room temperature (15–25 °C) to further stress the drug product. Bevacizumab-800CW manufactured by the UMCG under GMP conditions has a storage temperature of 2–8 °C with a shelf life of four years substantiated by in-house stability data presented in the product dossier. The high-temperature stress test induced protein aggregates, which is a common intrinsic particle encountered during routine production of parenteral protein drug products [13,14].

### 2.8. Evaluation of Qualification Test Sets for GMP Training and Qualification Purposes

The two GMP QTSs (Section 2.6) were implemented in the GMP training and qualification procedures with a change control strategy. GMP training and qualification procedures were conducted according to the in-house standard operating procedures of the UMCG. For the evaluation of the GMP QTSs and for comparison with the previous GMP training and qualification procedures using manufacturing rejects, four initial qualification and two requalification procedures were conducted with the in-house developed GMP QTSs. The experience of visual inspection personnel and the hospital pharmacist involved in the GMP training and qualification procedures were discussed and summarized. The focus of this discussion was whether the two developed GMP QTSs were a significant improvement and the factors attributing to this.

## 3. Results

### 3.1. Literature Search

On 28 December 2024, the searches yielded 320 (Pubmed), 634 (Embase), and 3631 (Web of Science) hits. The literature search did not reveal any publications from the databases describing an adaptable QTS manufacturing protocol for personnel qualification. However, two review articles [9,15] were identified that outlined certain considerations and specifications that contribute to defining an optimal QTS. Furthermore, one research article was identified that described a QTS manufacturing process [16].

The optimal QTS must meet a number of specifications (Table 1). First, the QTS must be stable (multiple years) and withstand repeated handling over time. Second, the number of QTS containers should ideally be representative of a routine visual inspection procedure to optimally train and qualify personnel. Third, the content of the QTS containers should be fully representative of the products encountered during routine productions since the physio-chemical characteristics of the content (e.g., viscosity, surface tension, turbidity, and colored solution) can have an impact on the motion of the content and the particulate matter. Fourth, the QTS should contain a wide range of defective containers that are representative of the defects encountered during routine production (e.g., small/large particles, fibers, glass shards, and mechanical damage), especially since these particulates interact differently with light and, therefore, the detection probability may differ. Fifth, the QTS should also contain defect-free containers, which aid in training personnel not to erroneously reject acceptable products, thereby maintaining inspection accuracy. Finally, the QTS should be adaptable and expandable with experience and based on visual inspection trend analysis data to further optimize personnel training and qualification.

Although the literature search did not reveal any publications describing an adaptable QTS manufacturing protocol, only one study was identified that described a QTS manufacturing process. This study used spherical cross-linked polystryrene divylbenzene beads with different diameters (40–180 µm) to manufacture a QTS. However, several limitations of this method were identified [16].

First, the authors noted that the beads were spherical and that the motion characteristics as well as light interaction of these beads did not always represent the wide range of defects that are encountered during routine productions. Furthermore, the beads could not always be readily dispersed, but either floated (‘floaters’) on the meniscus of the fluid or sticked to the sides of the containers. Consequently, personnel could not always identify these containers as defective units, which made training and qualification procedures challenging since it was not always evident whether personnel truly missed these defects or that the defects were simply not observable due these phenomena. These phenomena are likely the result of poor wettability and low surface energy of the beads [17]. Lastly, the proposed method was not adaptable and the QTS could not be expanded based on experience and visual inspection trend analysis data since the QTS only contained beads [16]. Taken together, we considered that this method did not comply with the specifications and requirements of an ideal and adaptable QTS for personnel training. Therefore, we continued with further research to develop an adaptable QTS protocol complying with the specifications listed in Table 1.

Common guidelines, including EU GMP Annex 1, FDA, PDA, USP, BP, and Ph. Eur. were reviewed and the information about QTSs was summarized [1,6,7,8,10,12]. According to all reviewed guidelines, 100% of the manufactured drug product batch must undergo visual inspection to ensure quality and safety. Defects found during these inspections should be categorized as critical, major, or minor [7,12]. Critical defects present a potential hazard capable of causing permanent injury and may compromise the integrity of the vial, rendering the product unsuitable for human use. Major defects, while not posing a risk of permanent injury, are likely to be noticeable to the user and may alter the usability of the drug product. Minor defects have no impact on user safety or product functionality but are typically of cosmetic nature [12].

To ensure operator competence, annual requalification is mandated according to the EU GMP Annex 1, including a visual acuity test [1,7]. Training requirements also specify that operators should be thoroughly trained in defect recognition, particularly in identifying particulate contamination [7,10]. The qualification procedure should be designed to reflect the actual production environment, incorporating relevant features such as container type, inspection duration, and batch size to accurately represent production conditions [7,9,10].

Additionally, a commercial QTS can be procured, or QTS units may be collected from manufacturing rejects encountered during routine productions. An in-house manufactured QTS is also permissible if needed to meet specific testing conditions [7].

Different methods are available for vial coding/decoding, including labeling, numbering, or using QR codes or UV markers, though each method has distinct limitations. For instance, QR codes require specialized scanning equipment, which can be costly and labor-intensive for decoding, while manual and observable numbering (e.g., using labels), although straightforward, may be subject to error if not updated regularly and do not provide fully blinded qualification procedures [7].

### 3.2. Survey Hospital Pharmacy Compounding Facilities

To evaluate existing procedures for personnel qualification involved in the visual inspection of parenteral drug products, we conducted a survey in which we contacted six hospital pharmacy compounding facilities in the Netherlands to gather insights into their practices. The survey involved the participation of five different hospital pharmacy compounding facilities besides the UMCG compounding facility. These included two tertiary and three secondary hospitals, all in possession of a GMP manufacturing license. The facilities produce various products that are visually inspected (e.g., ready-to-administer (RTA) syringes, bags for intravenous infusion, containers, and ampoules). All hospital pharmacy compounding facilities responded, and four (besides the UMCG) agreed to publish the synopsis of the observed methodologies, as detailed in Table 2. The results from the UMCG compounding facility describe the situation before development and implementation of the GMP QTSs.

It was observed that in-house developed QTSs were commonly used in the compounding facilities. However, there exists variability in the used excipients, manufacturing processes, and the QTS batch size across different facilities. Additionally, the acceptance criteria differ between the compounding facilities.

### 3.3. Semi-Structured Personnel Interviews

The semi-structured interview with GMP personnel revealed an overview of typical defects encountered during visual inspection of parenteral drug products. Among the visible particles encountered, fibers emerged as the most prevalent. Glass particles were found with lower frequency, and defects such as rubber particles from the stoppers were even rarer. Further, small particles such as aggregates or agglomerates were seldom detected.

Typical defect characteristics after gently swirling or inverting drug product containers were categorized on the basis of size (large or small), motion (fast, slow, or ‘cloudy’), ease of dispersion (readily or difficult), sedimentation rate (fast or slow), and interaction with light (does or does not reflect light). The characteristics of small particulate matter were classified as small spheres that are easy to disperse with a fast, sometimes ‘cloudy’ motion and slow sedimentation rate that do not reflect light readily. Fiber characteristics were classified as easy to disperse thread-like particles that could either be large or small with a slow motion and sedimentation rate that do not reflect light readily. Rubber particle characteristics were classified as a relatively large pellet that is difficult to disperse with a slow motion and fast sedimentation rate that does not reflect light readily. Glass particle characteristics were classified as a relatively large particle (shard) that is difficult to disperse with a slow motion and fast sedimentation rate that reflects light readily.

To ensure an objective and fully blinded inspection procedure, personnel stated that the vials should ideally be coded with an invisible coding system, preventing inspectors from memorizing vial numbers. Additionally, the QTS should encompass a sufficient variety of defect types and vials with no defects to optimally train personnel for the range of defects typically encountered during routine GMP productions, next to rarer defects since these are more difficult to observe because these defects are not encountered frequently.

### 3.4. Pre-GMP Formulation Studies

Pre-GMP and formulation research evaluated a variety of defects to determine how these defects could be introduced under GMP conditions, focusing on pharmaceutical- and GMP-grade excipients to simulate representative defects. In different and subsequent sessions, the defects were improved based on the feedback provided by personnel, ensuring relevance and practical applicability.

The materials and defects selected during the pre-GMP formulations studies and their advantages and disadvantages are listed in Table 3. Glass and rubber were found suitable to simulate defects found during visual inspection procedures. Paper fibers were deemed unsuitable due to brittleness and instability in aqueous solution due to disintegration within several days. A paper pulp fiber can absorb 30% of its mass in water [18], aiding in the wetting of the fibers and subsequent disintegration in smaller, not readily observable particles. The fiber from a Sontara^®^ Micropure AP cloth consists of a mixture of cellulose and polyester, making it more stable in an aqueous solution. Also, this material does not release other (undefined) particles that could contaminate the solution. Therefore, a fiber from this material was selected and found to be suitable for the QTS.

The introduction of talc and MCC particles as defects was challenging. On the one hand, larger talc particles (agglomerates) immediately disintegrated in the solution, yielding not readily observable particles. On the other hand, MCC agglomerates initially appeared to be stable, but also completely disintegrated within several days. These excipients proved to be useful to simulate small particles but could not be used to simulate larger particles for an extended period of time. Despite this, both talc and MCC provided realistic small-particle characteristics valued by personnel and, thus, were selected to be included in the final GMP QTS to simulate small particulate matter.

To overcome the limitations of talc and MCC, other materials were tested, namely barium sulfate, magnesium stearate, sodium carbonate, and silicon dioxide. Magnesium stearate was too hydrophobic and could not be optimally processed in aqueous solutions. The amount of sodium carbonate and silicon dioxide used during the experiments dissolved/disintegrated in water and yielded vials with defects that could not be readily detected. However, barium sulfate appeared to be suitable to simulate particulate matter in aqueous solution. Barium sulfate did not dissolve and had characteristics similar to protein aggregates in parental drug products, which has also been described elsewhere [5]. Furthermore, serial dilutions of barium sulfate suspensions could be produced to simulate high- and low-concentration aggregate solutions with varying degrees of difficulty to detect by the personnel, which was considered desired for training purposes.

### 3.5. Manufacturing Qualificaiton Test Sets Under GMP Conditions

Based on the literature search, personnel interviews and feedback, and pre-GMP formulation studies (Figure 1), the developed manufacturing protocol was used to produce the final GMP QTSs complying with the specifications of an optimal QTS (Table 1). The pre-GMP formulation studies ensured that all selected defect types could be effectively tech transferred and reproduced in a GMP cleanroom setting. This approach allowed us to manufacture two GMP QTSs that simulated defects encountered during routine GMP productions while complying with GMP standards.

**Figure 3:**
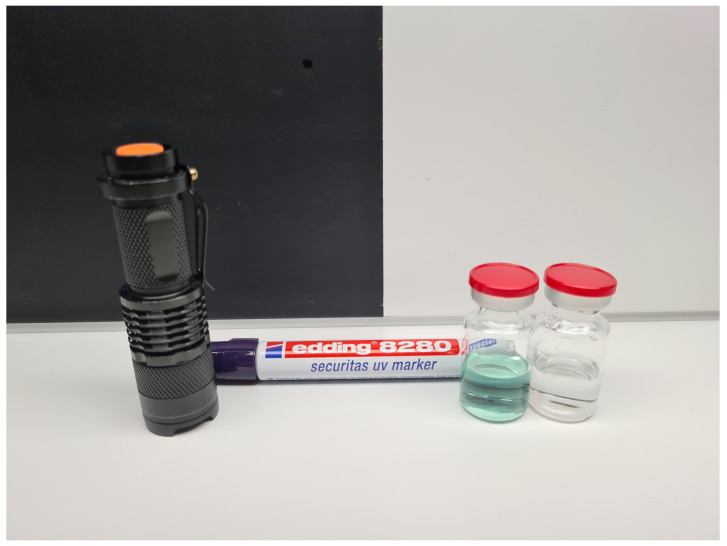
UV lamp and UV marker with two vials from the final GMP QTSs filled with either WFI + fluorescent dye IRDye 800CW (left vial, colored solution) or WFI (right vial, clear solution).

The final GMP QTSs both contained 100 glass containers manufactured in a LAF. Glass vials filled with WFI, capped with bromobutyl rubber stoppers and aluminum crimp sleeves, were used as containers. To address the needs of different product formulations, two QTSs were manufactured: one for clear solutions and another simulating colored solutions, specifically those similar to bevacizumab-800CW (Figure 3) [11]. The defect types introduced into these vials are detailed in Table 4 and the manufacturing protocol of the QTS containing the colored solution is given in the Supplementary Materials.

**Figure 4:**
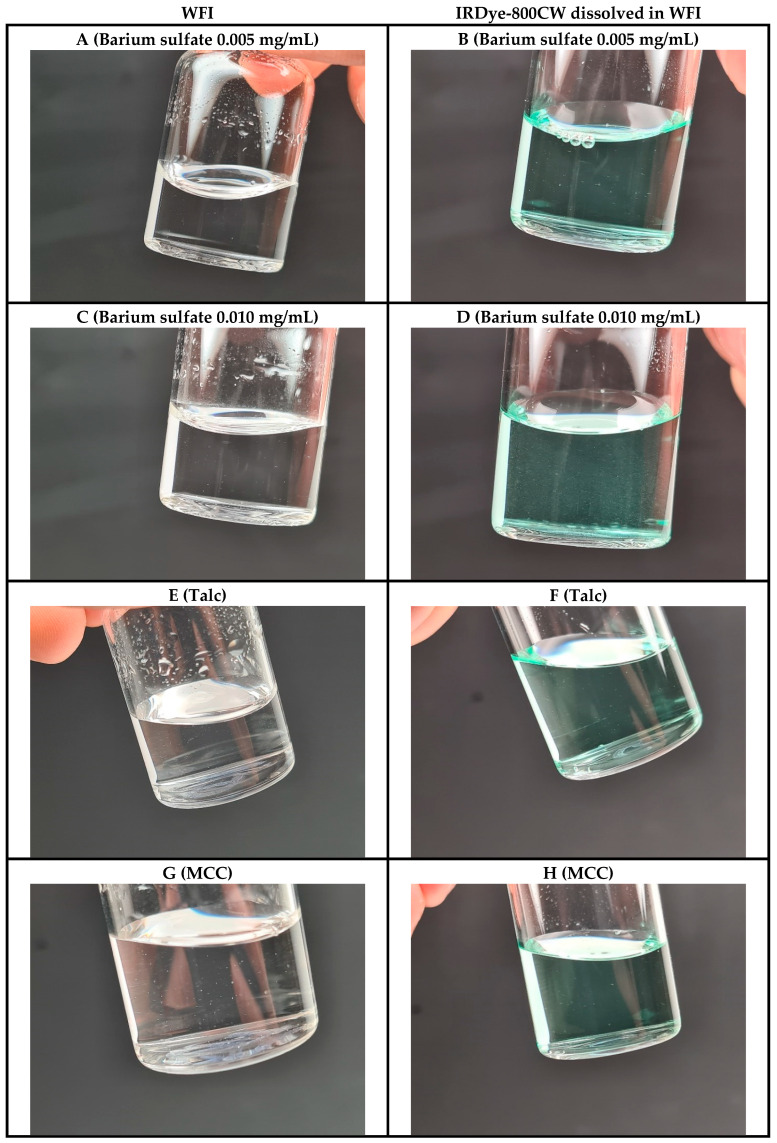
Representative images of the manufactured GMP QTSs containing either WFI (left, clear solution) or IRDye-800CW dissolved in WFI (right, colored solution) simulating small intrinsic and/or extrinsic particulate matter as defects. (**A**,**B**) Barium sulfate 0.005 mg/mL; (**C**,**D**) barium sulfate 0.010 mg/mL; (**E**,**F**) talc; (**G**,**H**) microcrystalline cellulose (MCC).

**Figure 5:**
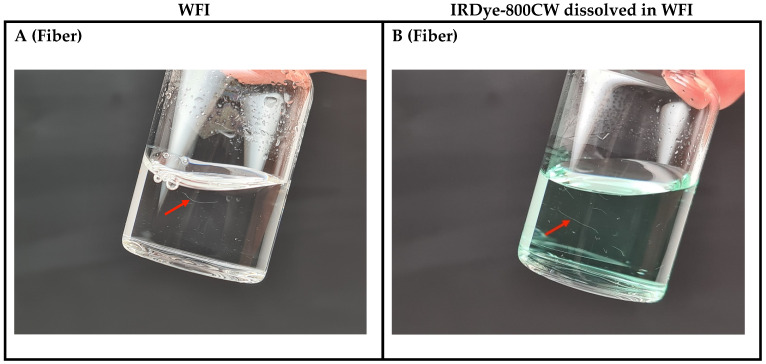

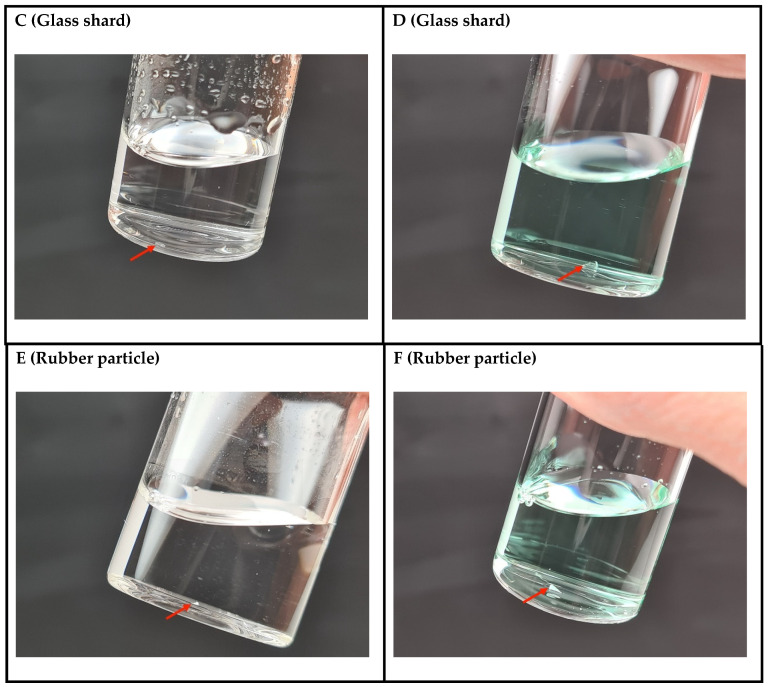
Representative images of the manufactured GMP QTSs containing either WFI (left, clear solution) or IRDye-800CW dissolved in WFI (right, colored solution) simulating either large intrinsic particulate matter or extrinsic particulate matter as defects. (**A**,**B**) Fibers; (**C**,**D**) glass shard; (**E**,**F**) rubber particle. The red arrows point at the particulate matter present in the vial.

The GMP QTSs included diverse defect materials—glass, rubber, fibers from Sontara^®^ cloth, talc, MCC, and barium sulfate—selected based on their ability to simulate defects encountered during routine GMP productions (Figure 4 and Figure 5). The inclusion of multiple defect types was selected to train and qualify personnel for a wide range of defects. The defects were categorized as critical, major, or minor.

Each container in the set was uniquely marked with a UV marker to bear either the number 1 (no defect) or 2–16 (defects), and this marking was designed to be easily discernible under a UV lamp (Figure 2). A comprehensive decoding table used by the trainer (a hospital pharmacist) of the personnel accompanied the set, providing a detailed description of the different defects along with their corresponding numbers for reference during the qualification procedure.

To ensure the validity of the introduced defects and the absence of defects in non-defective containers, a thorough four-eye consensus method was employed. This method was executed to guarantee that the defects were distinctly visible and that non-defective containers indeed were free of defects. The stability of the test set was validated over a two-year period using this consensus method.

### 3.6. Comparison Qualification Test Set Defects with Protein (Monoclonal Antibody) Aggregates

**Figure 6:**
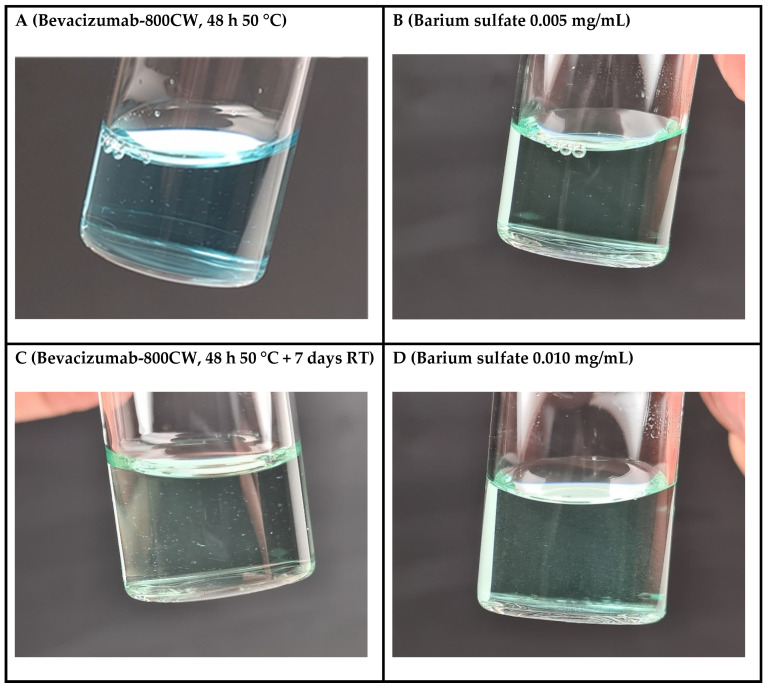
Barium sulfate containers from the GMP QTS simulated protein aggregates the best. (**A**) Bevacizumab-800CW stored at 50 °C for 48 h; (**B**) IRDye-800CW dissolved in WFI containing barium sulfate (0.005 mg/mL). (**C**) Bevacizumab-800CW stored at 50 °C for 48 h + 7 days at room temperature; (**D**) IRDye-800CW dissolved in WFI containing barium sulfate (0.010 mg/mL).

To assess whether the defects introduced into the GMP QTSs realistically simulated intrinsic particulate matter (e.g., protein aggregates), bevacizumab-800CW vials manufactured under GMP conditions were placed in an incubator for 48 h at 50 °C and at room temperature for an additional 7 days as a stress test to induce protein aggregates in the solution. After 48 h of storage at 50 °C, the bevacizumab-800CW vials contained detectable aggregates (Figure 6A), which showed similar particle characteristics (e.g., motion, particle size, and sedimentation rate) as the GMP QTS container containing a barium sulfate suspension of 0.005 mg/mL (Figure 6B). After an additional storage period of 7 days at room temperature, the bevacizumab-800CW vial contained more and visibly larger aggregates (Figure 6C), which showed similar particle characteristics as the GMP QTS container containing a barium sulfate suspension of 0.010 mg/mL (Figure 6D). Taken together, these results demonstrate that the barium sulfate suspension is suitable to simulate varying degrees of protein aggregates by using different barium sulfate concentrations.

### 3.7. Evaluation of Qualification Test Sets for GMP Training and Qualification Purposes

The developed QTSs manufactured under GMP conditions were used for GMP training and formal visual inspection qualification procedures. Personnel stated that the GMP QTSs were a significant improvement for training and qualification purposes compared to using manufacturing rejects. Factors attributed to this improvement were a fully blinded qualification procedure, the use of fully representative containers and solutions in a representative batch size, the wide range of defects, the possibility to use less/more challenging defects, and the possibility to include new defects based on routine GMP productions and manufacturing reject trend analysis. Personnel also stated that the wide range of introduced defects simulated the wide range of intrinsic and particulate matter encountered during GMP productions.

Based on the experience and evaluation of the hospital pharmacist involved in the GMP training and formal visual inspection qualification procedures, the GMP QTSs were seen as a significant improvement compared to QTSs using manufacturing rejects. Besides the abovementioned factors stated by personnel, a significant factor attributing to this improvement was using a fully qualified GMP QTS in which the defects and non-defects were confirmed by the consensus method (Section 3.5), which ensured the validity of the introduced defects and the absence of defects in non-defective containers. Therefore, all qualification results could be interpreted unambiguously, which is considered to be an improvement compared to qualification procedures using manufacturing rejects (discussed in Section 4).

## 4. Discussion

The present study describes the development and feasibility of an adaptable and optimal QTS for personnel involved in visual inspection procedures of parenteral drug products manufactured under GMP conditions in hospital pharmacy compounding facilities. Although personnel qualification is required by GMP guidelines, no adaptable manufacturing protocols or formal guidelines for the development of an optimal and in-house manufactured QTSs are available in the current literature, which possibly points at unstandardized visual inspection qualification procedures between different production sites. The survey results from the five Dutch hospital pharmacies indeed showed that the used QTSs, procedures, and acceptance criteria differed between the production sites. To standardize the visual inspection qualification procedure and use a QTS that is not only representative for the defects and containers encountered during routine GMP productions but also compliant with guidelines on visual inspection of parental drug products, we developed an adaptable manufacturing protocol for an in-house manufactured GMP QTS based on these guidelines and personnel interviews. As a proof-of-concept, two QTSs were manufactured under GMP conditions, showing the feasibility and adaptability of the protocol. To the best of our knowledge, this is the first study presenting these data, and our developed manufacturing protocol may aid compounding facilities in the standardization, training, and qualification of personnel involved in visual inspection procedures.

The literature search identified key elements and attributes for an optimal QTS and visual inspection procedures, which we considered as relevant for an adaptable QTS protocol [1,7,9]. The interviews with GMP personnel further supported these findings, indicating the practical relevance of these key elements. Therefore, these key elements and attributes were used for the development of our protocol [1,8,10,12].

The literature search also identified one study describing a QTS manufacturing process [16]. However, we considered this method unsuitable for the development of our protocol since it did not comply with the key elements and attributes for an optimal QTS.

The survey among hospital compounding facilities revealed considerable variability in the qualification procedures and used test sets. Although standardized processes and qualification procedures are desired within GMP compounding facilities to ensure a consistent level of training quality, no practical protocols or guidelines are currently available for visual inspection test sets or procedures. Our developed GMP QTS manufacturing protocol addresses this gap by providing an in-house and adaptable manufacturing protocol for GMP compounding facilities that can be readily implemented.

The adaptability of the manufacturing protocol is particularly beneficial for hospital pharmacy compounding facilities, which generally operate with fewer resources, manufacture smaller batch sizes, but have larger product ranges than a given pharmaceutical company. Therefore, the protocol can be easily adapted to manufacture QTSs relevant for different products (e.g., different fill volumes, colored/turbid solutions, different primary packaging, et cetera) at low costs without the need to amass sufficient manufacturing rejects, which is especially challenging for products with smaller batch sizes. Moreover, the wishes, experience of personnel involved in visual inspection procedures, and manufacturing reject trend analyses can be used to further optimize the test set, and by extension, the training, qualification, and inspection procedures.

Using manufacturing rejects to develop QTSs can lead to a biased and incomplete number of defects present in the test set since the composition is fully dependent on rejects encountered during routine productions [9]. In addition, the interpretation of the visual inspection qualification results may be challenging in case manufacturing rejects are used for the QTSs. During the UMCG qualification procedures using manufacturing rejects, some personnel stated that containers coded as defects could not be identified as such, and containers coded as non-defects were identified as defects. A possible reason for this discrepancy could be that the defect was either too challenging for the personnel or that the defect, initially present, disappeared over time; for instance, in case the particulate matter dissolved or fully disintegrated after an extended period of storage. Another reason could be that a defect was introduced over time in containers that initially did not contain any defects; for instance, container damage due to frequent handling or the precipitation of the active pharmaceutical ingredient or excipients over time. The question then arises: are the current, discrepant observed qualification results true or not? This makes unambiguous interpretation of the qualification results challenging.

For the developed QTSs, insoluble and not readily disintegrating materials were used to introduce stable defects that are presumed to be present for an extended period of time. Furthermore, the consensus visual inspection method was employed and ensured that all introduced defects were accurately identified as such and confirms that the containers that did not contain any defects were indeed identified as such. This procedure can be seen as the qualification of the test set itself with the four-eye method, which warrants its validity and can be periodically repeated (e.g., annually). In case the QTS does not (or no longer) meets the desired criteria, a new QTS can be manufactured by the compounding facility. Based on our own experience, the developed GMP QTSs are stable at room temperature for at least two years.

Since no manufacturing rejects are required for the developed GMP QTSs, these rejects from the visual inspection procedure can possibly be used for preclinical research or process optimization procedures of the manufactured drug products. For instance, manufacturing rejects containing extrinsic particulate matter (e.g., fiber or glass shard) cannot be administered to patients but can be used to develop, validate, or optimize analytical methods of the drug product, which is of particular interest for expensive drug products such as fluorescent-labelled monoclonal antibodies [19] or neoantigen vaccines [20].

Besides personnel qualification, the adaptable manufacturing protocol for a QTS could be employed for the process development and optimization of (semi-)automated visual inspection technologies. With the possibility to manufacture different batch sizes containing any desired defect in any desired frequency, these QTSs could be used to calibrate, qualify, and validate these technologies. Depending on the process parameters and required accuracy of the systems, the manufacturing protocol can be adapted accordingly to yield a QTS that is suitable for system qualification and validation. However, to the best of our knowledge, the qualification and validation of (semi-)automated visual inspection technologies with in-house manufactured QTSs have not been described in the literature. Therefore, the application and feasibility of our developed QTS for (semi-)automated systems needs to be investigated.

Although we used a mixed methods study design, there are several limitations of the present study. First, the survey sample size was limited to five large Dutch hospital pharmacy compounding facilities. Therefore, the survey result did not present data of centralized compounding facilities or other, smaller hospital pharmacy compounding facilities in the Netherlands or abroad. Second, only experienced personnel of the UMCG were interviewed, and these data were used for the development and optimization of the QTS formulations. Third, for the formulation studies and the proof-of-concept, we only focused on clear and colored aqueous solutions in clear primary containers. However, it is expected that the motion characteristics as well as visibility of particulate matter differ in viscous or turbid solutions packaged in opaque or colored (e.g., amber) primary containers [9,15]. Finally, for proof-of-concept, we manufactured two GMP QTSs with a limited amount of containers containing small particulate matter (talc, MMC, 0.010 mg/mL, and 0.005 mg/mL). To further challenge personnel, serial dilutions of these formulations can be made with a resulting lower particle count, which yield QTS containers that are more challenging to detect during qualification procedures. These limitations should be considered and taken into account for the development of in-house manufactured QTSs by compounding facilities using the provided protocol (Supplementary Materials). 

In conclusion, this study presents the development of a practical, feasible, and adaptable manufacturing protocol for a QTS for the qualification of personnel involved in the visual inspection of parental drug products manufactured under GMP conditions. Due to its adaptability, the qualification test set can be expanded and further customized and optimized based on specific wishes, personnel experiences, and manufacturing reject trend analyses. Together, our developed QTS manufacturing protocol has been proven to be a valuable addition to process standardization, training, and qualification of personnel.

## Figures and Tables

**Table 1:** Specifications, requirements, and considerations for an optimal QTS for personnel training and qualification procedures.

Specification	Requirement and Consideration
Stability	Stable for years; withstand repeated handling over time
Number of containers	Fully representative of routine production; approximately 15–20% defective containers
Content containers	Fully representative of routine production; e.g., turbidity, viscosity, colored solutions, surface tension; physio-chemical characteristics may impact motion and light interaction of particulate matter
Primary packaging	Fully representative of routine production; e.g., colored glass, opaque, syringe, ampoules, vials; ease of dispersion of particulates in different containers may differ
Defects	Fully representative of routine production; particles, fibers, glass shards, mechanical damage; different particles interact differently with light; defects should be adaptable to manufacture less/more challenging containers for personnel training and qualification
Defect-free containers	Aids in training personnel not to erroneously reject acceptable products
Adaptability	Should expand based on experience and visual inspection trend analysis data; new defects may be introduced

**Table 2:** Summary of results from the survey for hospital pharmacy compounding facilities in the Netherlands. Abbreviations: VAT: visual acuity testing. Opth.: ophthalmologist. QTS: qualification test set. MCC: microcrystalline cellulose. WFI: water for injection.

	UMCG	Hospital A	Hospital B	Hospital C	Hospital D
**Level of healthcare**	Tertiary care	Secondary care	Secondary care	Tertiary care	Secondary care
**Personnel qualification**	VAT via opht. and test set	VAT via opht. and test set	VAT via opht. and test set	VAT via opht. and test set	VAT via opht. and test set
**Frequency of qualification**	Annually	Annually	Annually	Annualy VAT, Once per two years test set	Annually
**Development of QTS**	Obtained from manufacturing rejects of routine productions	In-house developed Only particles	Commercial QTS	Obtained from manufacturing rejects of routine productions	In-house developed Only particles
**Number of containers in the QTS**	100	30	100	50	30
**Composition of QTS**	No distinction between types of particles	MCC	Glass, fibers, incorrect ampoule heads, volume variations	No distinction between types of particles	12.5 mg/L MCC and tap water
**Acceptance criteria**	Not more than 0 critical, 1 major, 5 minor defects should be missed. Incorrect rejection: ≤15%.	100% of all defects must be identified. Incorrect rejection: maximum of 2 syringes.	Three QTS’s are visually inspected. ≥75% of all defects must be identified and ≥70% of defects must be identified per individual QTS. Incorrect rejection: ≤10%.	≥70% of all defects must be identified. Initial failure: repeat with double QTS, until the employee is proficient.	100% of all defects must be identified. Incorrect rejection: in WFI, 7 out of 9 is allowed.
**Decoding of containers**	Decoding table with visual numbers.	Decoding table with visual numbers. The numbers on the vials are replaced after qualification of 2–3 employees and when a new QTS is developed.	UV lamp.	Decoding table with visual numbers. A unique subset is used for each qualification. Employees are not informed which containers were rejected correctly.	UV lamp.

**Table 3:** Defect types and materials used to simulate different intrinsic and extrinsic particles with their advantages and disadvantages.

Defect Materials	Simulates	Characteristics in Solution	Advantages	Disadvantages
Talc	Particulate mater (intrinsic, extrinsic)	Small spheres Easy to disperse Slow sedimentation rate	Available in pharmaceutical grade, compatible in cleanroom areas Realistically simulates intrinsic and extrinsic particles	Disintegrates in aqueous solution Particle size may decrease over time
Microcrystalline cellulose (MCC)		Small spheres Easy to disperse Slow sedimentation rate	Available in pharmaceutical grade, compatible in cleanroom areas Realistically simulates intrinsic and extrinsic particles	
Barium sulfate		Small spheres ‘Cloudy’ motion Easy to disperse Slow sedimentation rate	Available in pharmaceutical grade, compatible in cleanroom areas Insoluble in water Serial dilution of known concentration can be produced to challenge personnel Realistically simulates protein aggregates	None observed
Magnesium stearate		Large clumps	Available in pharmaceutical grade, compatible in cleanroom areas	Too hydrophobic to properly process in aqueous solutions
Silicon dioxide		Not visible	Available in pharmaceutical grade, compatible in cleanroom areas	Used amount either dissolved in aqueous solution or particles were too small to visually observe
Sodium carbonate		Not visible	Available in pharmaceutical grade, compatible in cleanroom areas	
Glass, chipped from Pasteur capillary pipette	Glass shards (intrinsic)	Large particle Reflects light Difficult to disperse Fast sedimentation rate	Available in pharmaceutical grade, compatible in cleanroom areas Realistically simulates intrinsic particles from glass vial used during routine production	Chipping glass may generate fine glass particles in cleanroom area
Rubber, cut from bromobutyl stopper	Rubber particle(intrinsic)	Large pellet Difficult to disperse Fast sedimentation rate	Available in pharmaceutical grade, compatible in cleanroom areas Realistically simulates intrinsic particles from bromobutyl stopper used during routine production	Challenging to cut a fine particle in LAF cabinet with tweezers and scalpel
Fiber, sterile cloth	Fiber (extrinsic)	Small or large thread-like particle Easy to disperse Slow sedimentation rate	Available in pharmaceutical grade, compatible in cleanroom areas Realistically simulates extrinsic particles from sterile mat or cleaning/disinfection cloth used during routine production	Fiber may eventually disintegrate over time, making the defect undetectable Challenging to cut a fine fiber in LAF cabinet with tweezers and scissors
Fiber, paper towel		Small thread-like particle Easy to disperse Slow sedimentation rate Fully disintegrates	Widely available Easy to process	Unstandardized quality, incompatible with higher-grade cleanroom areas Tearing generates airborne particles Quickly and fully disintegrates, making the defect undetectable
Dented aluminum crimp	Mechanical damage primary packaging	Not applicable	Realistically simulates mechanical damage encountered during routine production Defects can be introduced after production process outside LAF cabinet Different damages can be introduced to simulate minor (cosmetic) or major (packaging integrity) damages to challenge personnel	None observed
Chipped plastic cap protecting vial septum	Mechanical damage primary packaging	Not applicable		

**Table 4:** The composition of the final GMP QTSs composed of 100 containers.

Defect	Simulates	Defect Classification	Frequency
Dent crimp seal	Cosmetic damage, mechanical stress	Minor	2%
Damaged plastic cap, rubber stopper exposed	Critically damaged product, loss of rubber septum sterility	Critical	2%
Rubber particle	Extrinsic particulate matter from bromobutyl rubber	Major	1%
Glass shard	Extrinsic particulate matter from glass vial	Major	2%
Small particulate matter	Extrinsic or intrinsic (protein aggregates, drug precipitates) particulate matter	Major	4% ^a^
Fibers	Extrinsic particulate matter from sterile mat or cleaning/disinfection cloth	Major	4%
No defects	Drug products with no defects that should not unjustly be rejected	Not applicable	85%

^a^ One container with MCC, one container with talc, one container with 0.005 mg/mL barium sulfate, and one container with 0.010 mg/mL barium sulfate. See also the Supplementary Materials.

## Data Availability

The data are contained within the article.

## Supplementary Materials

The following supporting information can be downloaded at https://www.mdpi.com/article/10.3390/pharmaceutics17010074/s1, Manufacturing protocol for the GMP QTS colored with fluorescent dye IRDye 800CW.

## Appendix B. Questionnaire Survey National Hospital Pharmacy Compounding Facilities

Dear colleague,

Currently, we are in the process of developing and manufacturing a standardized qualification test set for the GMP visual inspection qualification procedures of personnel. We intend to manufacture the qualification test set in our compounding facility under GMP conditions, including containers with defects and particulate matter. We intend to manufacture the defective units by adding known particles (fibers, hairs, glass, particles, etc.).

Therefore, we are curious about the strategies employed by other hospital pharmacies. Could you please answer the following questions:

1. How do you qualify personnel involved in the visual inspection of parenteral drug products?
2. How do you develop the qualification test set consisting of containers with and without particles/defects?
3. How many defective containers does your qualification test set contains?
4. What are the particles/defects in the qualification test set?
5. What is your experience regarding the visual inspection qualification procedures of personnel?
6. What are your acceptance criteria for qualifying/disqualifying personnel, e.g., maximum number of missed defects, maximum number of compliant units that are rejected, etc.?
7. Is it possible to review your qualification procedure (confidential, of course)? If desired, we can also send ours.

We hope that you can assist us and look forward to your responses.

Kind regards,

## Appendix C. Questionnaire for GMP Personnel Regarding the Visual Inspection

During the interview, detailed follow-up questions were asked to obtain more information about common particles and defects encountered during routine production.

1. Could you explain what visual inspection of parental drug products is?
2. Please describe the visual inspection procedure in detail.
3. Is there a four-eye check during the visual inspection?
4. What are common particles encountered during the visual inspection procedure?
5. Is the origin of these particles always clear or known?
6. What are particles that are difficult to detect during the visual inspection procedure?
7. What are common defects that are found during the visual inspection procedure, such as packaging integrity issues or (cosmetic) damages to the packaging?
