# Correction: van den Born-Bondt et al. Development of an Adaptable Qualification Test Set for Personnel Involved in Visual Inspection Procedures of Parenteral Drug Products Manufactured Under Good Manufacturing Practice Conditions in Hospital Pharmacy Compounding Facilities. *Pharmaceutics* 2025, *17*, 74

**DOI:** 10.3390/pharmaceutics17050564

**Published:** 2025-04-25

**Authors:** Tessa van den Born-Bondt, Harmen P. S. Huizinga, Koen R. Kappert, Hans H. Westra, Jacoba van Zanten, Herman J. Woerdenbag, Jacoba M. Maurer, Bahez Gareb

**Affiliations:** 1 Department of Clinical Pharmacy and Pharmacology, University Medical Center Groningen (UMCG), 9713 GZ Groningen, The Netherlands; 2 Department of Pharmaceutical Technology and Biopharmacy, Groningen Research Institute of Pharmacy (GRIP), University of Groningen, Antonius Deusinglaan 1, 9713 AV Groningen, The Netherlands

## Error in Figure

In the original publication [1], there was a mistake in Figure 4A as published. Figure 4A,C were unintentionally duplicated (identical images) and the corrected Figure 4 appears below. The authors state that the scientific conclusions are unaffected. This correction was approved by the Academic Editor. The original publication has also been updated.

## Figures and Tables

**Figure 4:**
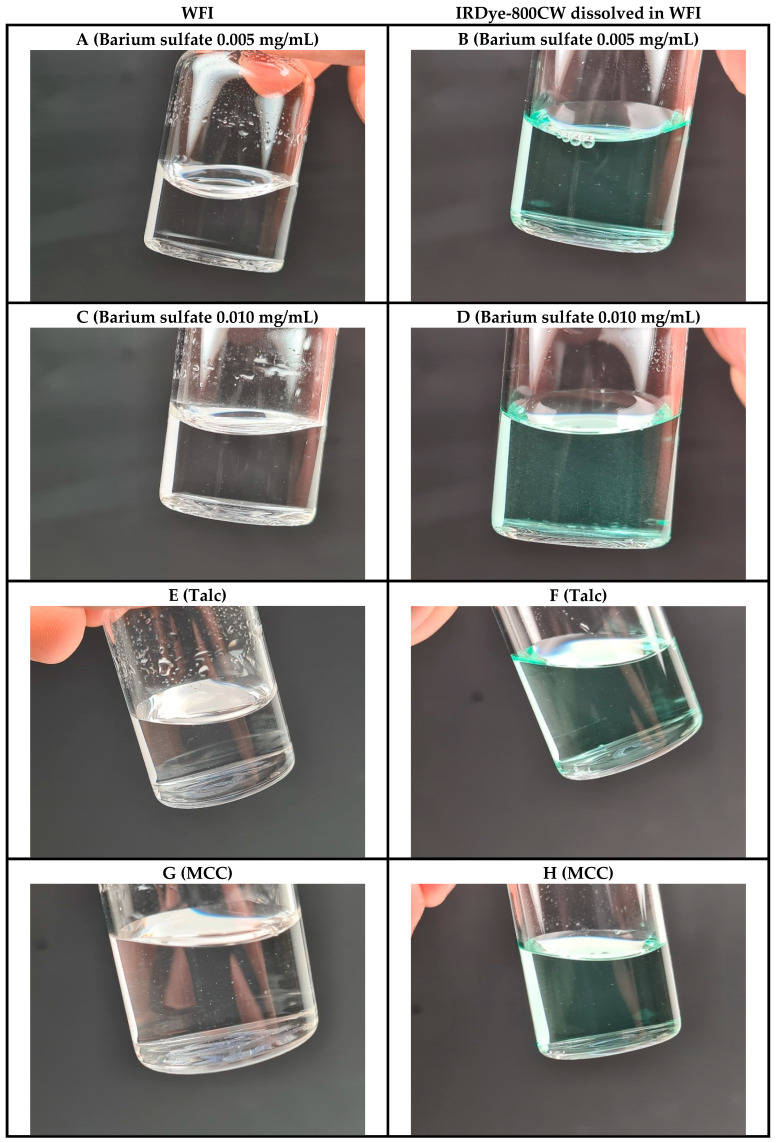
Representative images of the manufactured GMP QTSs containing either WFI (left, clear solution) or IRDye-800CW dissolved in WFI (right, colored solution) simulating small intrinsic and/or extrinsic particulate matter as defects. (**A**,**B**) Barium sulfate 0.005 mg/mL; (**C**,**D**) barium sulfate 0.010 mg/mL; (**E**,**F**) talc; (**G**,**H**) microcrystalline cellulose (MCC).
