# Supplementary material for: Development of an Adaptable Qualification Test Set for Personnel Involved in Visual Inspection Procedures of Parenteral Drug Products Manufactured Under Good Manufacturing Practice Conditions in Hospital Pharmacy Compounding Facilities
Source: Pharmaceutics. 2025 Jan 7;17(1):74. doi: 10.3390/pharmaceutics17010074 (PMC11768810; doi:10.3390/pharmaceutics17010074)
Supplement: Supplementary file 1 [file pharmaceutics-17-00074-s001.zip › pharmaceutics-3398091-supplementary.pdf]

PROTOCOL AUTHORISATION

Job title / function	Name	Date and signature

BATCH APPROVAL

Job title / function	Name	Date and signature

Product released	Yes/no
Date and signature	

Deviation description (if applicable)	Deviation number (if applicable)

TABLE OF CONTENTS

INTRODUCTION 3
GENERAL NOTICE 3
BACKGROUND 3
PROCESS INSTRUCTIONS 4
CHANGE LOG 4
OPERATOR(S) 4
LIST OF MATERIALS 5
APPARATUS AND UTENSILS 7
1. PREPARING THE 800CW NHS ESTER SOLUTION 8
2. PREPARING THE DEFECTS OF THE QTS 9
4. PREPARATION OF THE B GRADE CLEANROOM 14
5. ASEPTIC FILLING OF VIALS WITH IRDYE 800CW SOLUTION 15
APPENDIX A: LABEL RECONCILIATION 21
APPENDIX B: NOTES 22

INTRODUCTION

This protocol describes the manufacturing process of a colored qualification test set (QTS) used for training and qualification procedures. The QTS must accurately represent defects encountered during routine GMP productions of clear and greenish colored solutions, such as that of fluorescent-labelled monoclonal antibodies labelled with IRDye 800CW.

The QTS manufacturing process consists of the following procedures:

- Preparing a IRDye 800CW NHS ester solution
- Preparing the "defects"
- Transferring the IRDye 800CW NHS ester solution into a Flexboy bag
- Filling the vials with IRDye 800CW NHS ester solution including defects (15x) and without defects (85x)

GENERAL NOTICE

During this production process the regular disinfectants and cleaning agents can be used.

BACKGROUND

The compounding facility manufactures parenteral drug products under GMP conditions. These drug products should be visually inspected according to Ph. Eur. 2.9.20 by trained and qualified personnel. To train and qualify personnel, QTS's can be used. The QTS should be representative and contain defects encountered during routine production. Since the compounding facility manufactures clear and greenish colored fluorescent-labelled monoclonal antibodies, personnel should be trained with a QTS containing clear and greenish solutions in the primary packaging that contain defects and no defects. The QTS will contain the defects listed in Table 1.

Table 1: Defects and their corresponding vial numbers in the QTS. Vials containing no defects are numbered "1".

<table>
<tr><th>Vial #</th><th>Defect</th><th>Frequency per 100</th><th colspan="2">Critical / major / minor</th></tr>
<tr><td>1</td><td>No defects</td><td>85</td><td colspan="2">Not applicable</td></tr>
<tr><td>2 and 3</td><td>Indentation crimp cap</td><td>2</td><td colspan="2">Minor</td></tr>
<tr><td>4 and 5</td><td>Broken crimp cap (septum exposed)</td><td>2</td><td colspan="2">Critical</td></tr>
<tr><td>6</td><td>Rubber</td><td>1</td><td colspan="2">Major</td></tr>
<tr><td>7 and 8</td><td>Glass</td><td>2</td><td colspan="2">Major</td></tr>
<tr><td>9</td><td>Talc</td><td>1</td><td colspan="2">Major</td></tr>
<tr><td>10</td><td>Microcrystalline cellulose</td><td>1</td><td colspan="2">Major</td></tr>
<tr><td>11</td><td>Barium sulphate (0,010 mg/ml)</td><td>1</td><td colspan="2">Major</td></tr>
<tr><td>12</td><td>Barium sulphate (0,020 mg/ml)</td><td>1</td><td colspan="2">Major</td></tr>
<tr><td>13, 14 and 15</td><td>Small fiber</td><td>3</td><td colspan="2">Major</td></tr>
<tr><td>16</td><td>Large fiber</td><td>1</td><td colspan="2">Major</td></tr>
<tr><td rowspan="3"></td><td rowspan="3">Total:</td><td rowspan="3">15</td><td>Critical</td><td>2</td></tr>
<tr><td>Major</td><td>11</td></tr>
<tr><td>Minor</td><td>2</td></tr>
</table>

PROCESS INSTRUCTIONS

The preparation of the defects, the IRDye 800CW NHS ester and the transfer to the Flexboy bag is performed in a laminar air flow cabinet. The filling of the vials is done in a biosafety cabinet.

CHANGE LOG

-Not applicable. Version 1.

OPERATOR(S)

Operator(s)	Initials

LIST OF MATERIALS

Raw materials	Article number supplier	Manufacturer	Batch number	Expiry date	Initials	4-eye
IRDYE 800CW NHS ESTER IN DMSO 5 MG/ML (3 VIALS of 750 µL)						
WATER FOR INJECTION (WFI) 1000 ML						
BARIUM SULPHATE						
MICROCRYSTALLINE CELLULOSE (MCC), 1000 gr						
TALC SIZE 500 gr						

Materials and disposables	Amount	Article number	Manufacturer	Batch number	Expiry date	Initials
GLAS VIAL EASY FILL CLEAR 24.0X45.0 10R TRAY	2 trays					
OMNIFLEXPLUS 20 MM STOPPER, GREY, STERILE BAG	130 pcs					
CRIMP CAP RED CTO 20 MM, STERILE BAG	130 pcs					
FILTER MILLEX GP 0,22UM 33MM	3					
CONICAL TUBE PP 30X115MM 50ML SCREW CAP	1					
SCISSORS	1					
PLIERS	1					
HOSE LS/15	1					
HOSE SET ASEPTIC FILLING 6 ML	1					
PASTEUR PIPETTE	1					
CLEANROOMWIPE STERILE , SONTARA MICROPURE AP, 224X224MM, W	1					
VOLUMETRIC FLASK 100 ML	1					
BEAKER 1000 ML	2					
STERICAN DISPOSABLE CANULE 4665120 1,2X40MM	2					

APPARATUS AND UTENSILS

Name	Serial number	Room number
BALANCE SARTORIUS SECURA 213-1CEV		
BIOSAFETY CABINET		
PERISTALTIC DOSING PUMP; WATSON MARLOW		
AP/CP2000 SEMI-AUTOMATIC CRIMPING TOOL; BODY		
AP/CP2000 SEMI-AUTOMATIC CRIMPING TOOL; HEAD		
PIPETTE P1000		
PERISTALTIC PUMP MASTERFLEX		
UV MARKER EDDING 8280 SPECIAL		
UV FLASHLIGHT WITH ZOOM		

1. Preparing the 800CW NHS ester solution

STEP	INSTRUCTIONS	REQ.	RESULT	INITIALS OP. 1	INITIALS OP. 2
1.01	**Preparing the 800CW NHS ester solution**				
	Remove the crimp cap from the 1L WFI bottle but leave the stopper on the bottle.				
	Transfer 2 mL IRDye 800CW NHS ester (5 mg/mL) with a pipette to the 1000 mL WFI bottle.	2 mL			
	Carefully homogenize the solution.				

2. Preparing the defects of the QTS

STEP	INSTRUCTIONS	REQ.	RESULT	INITIALS OP. 1	INITIALS OP. 2
2.01	**Print labels for the following steps**<br>The defects and numbers are listed in Table 1.<br>Step 2.02 No. 7 and 8<br>Step 2.03 No. 13, 14, 15 and 16<br>Step 2.04 No. 10<br>Step 2.05 No. 11 and 12<br>Step 2.06 No. 6<br>Step 2.07 No. 9<br>Step 2.08 No. 2 and 3<br>Step 2.09 No. 4 and 5<br><br>Example label: Batch no.: yyMdd-xxx **Number X**<br>Used label:				
2.02	**Preparing the glass shards (2x)**<br><br>**Glass**:<br>Break a glass Pasteur pipette inside a Micropure AP non-woven wipe. Select a small shard using pliers. Transfer the shard to the vial. Close the vial immediately using a Omniflexplus stopper. Repeat the above process. Mark the vials with a temporary label. The second operator numbers the vial (Table 1)				
2.03	**Preparing small fibers (3x) and large fibers (1x)**<br><br>**Small fiber:**<br>Carefully tear a Micropure AP non-woven wipe. Use pliers to select a fiber. Cut off the protruding fiber ends on both sides of the pliers using a scissor.				

STEP	INSTRUCTIONS	REQ.	RESULT	INITIALS OP. 1	INITIALS OP. 2
	The fiber is now the size plier's tip. Transfer the fiber to the vial. Close the vial immediately using a Omniflexplus stopper. Repeat the above process two more times. Mark the vials with a temporary label. The second operator numbers the vials (Table 1).<br><br>**Large fiber:**<br>Carefully tear a Micropure AP non-woven wipe. Use pliers to select a fiber. The fiber should be two times larger than the small fiber. Transfer the fiber to the vial. Close the vial immediately using a Omniflexplus stopper. Mark the vial with a temporary label. The second operator numbers the vials (Table 1).				
2.04	**Preparing the microcrystalline cellulose solution (1x)**				
	**Microcrystalline cellulose:**<br>Transfer a small part of the IRDye 800CW NHS ester solution to a beaker so that the beaker bottom is completely covered with the solution. Add microcrystalline cellulose and swirl briefly. Use a spatula to take a large agglomerate from the solution. Then use a needle to select an isolated, smaller agglomerate from the spatula tip. Transfer this small agglomerate to the vial. Close the vial immediately using a Omniflexplus stopper. Mark the vial with a temporary label. The second operator numbers the vials (Table 1).				
2.05	**Preparing 0.010 mg/ml (1x) and 0.020 mg/ml (1x) barium sulphate solutions**				
	**Stock solution barium sulphate:**<br>Weigh 100 mg barium sulphate and suspend this in 100 ml IRDye 800CW solution in a 100 ml volumetric flask.<br><br>Add the weighing report as an appendix.				

STEP	INSTRUCTIONS	REQ.	RESULT	INITIALS Op. 1	INITIALS Op. 2
	**0.010 mg/mL:** Transfer 50 µL to a vial using a Gilson pipette. Close the vial immediately using a Omniflexplus stopper. Mark the vial with a temporary label. The second operator numbers the vials (Table 1).<br><br>**0.020 mg/mL:** Transfer 100 µL to a vial using a Gilson pipette. Close the vial immediately using a Omniflexplus stopper. Mark the vial with a temporary label. The second operator numbers the vials (Table 1).				
2.06	**Preparing the rubber particle (1x)**				
	**Rubber:** Cut off a small piece of an Omniflexplus stopper. Select a small piece of rubber using pliers. Transfer the rubber to a vial. Close the vial immediately using a Omniflexplus stopper. Mark the vial with a temporary label. The second operator numbers the vials (Table 1).				
2.07	**Preparing the talc solution (1x)**				
	**Talc:** Transfer a small part of the IRDye 800CW NHS ester solution to a beaker so that the beaker bottom is completely covered with the solution. Add talc without mixing. Use a spatula to take a large agglomerate from the solution. Then use a needle to select an isolated, smaller agglomerate from the spatula tip. Transfer this small agglomerate to the vial. Close the vial immediately using a Omniflexplus stopper. Mark the vial with a temporary label. The second operator numbers the vials (Table 1).				
2.08	**Denting the crimp cap (2x)**				
	After the vials are filled with the solution, dent the crimp cap of two vials using pliers. Mark the vial with a temporary label and number (Table 1)				

STEP	INSTRUCTIONS			INITIALS	
		REQ.	RESULT	OP. 1	OP. 2
2.09	**Breaking / damaging vial cap (exposed septum) (2x)**<br><br>After the vials are filled with the solution, use the tip of plier to carefully break / damage the vial cap so that the septum is partially exposed. Mark the vial with a temporary label and number (Table 1).				
2.10	**Filling vials with the 800CW NHS ester solution**<br><br>Transport the remaining IRDye 800CW NHS ester solution and the vials containing the defects to the B-grade cleanroom.				

3. Transferring the IRDYe 800CW NHS ester solution to Flexboy bag

STEP	INSTRUCTIONS	REQ.	RESULT	INITIALS OP. 1	INITIALS OP. 2
3.01	**Pump the IRDye 800CW NHS solution into a 3L Flexboy bag according to the SOP**<br><br>Add one end of the tube to the container containing the 800CW NHS ester solution and attach the other end to the inlet of the Sartopore 2 filter on the Flexboy bag.<br><br>Insert the tubing set into the Masterflex pump and make sure both ends of the tubing set, the container of 800CW NHS ester solution and the Flexboy bag remain in the LAF cabinet.<br><br>Turn on the pump and make sure that the solution is being transferred to the Flexboy bag. Continue pumping until all of the solution is transferred to the Flexboy bag.				
3.02	**Completing the IRDye 800CW NHS ester solution preparation**<br><br>Wrap the Flexboy bag containing the IRDye 800CW NHS ester solution in aluminum foil and transport the bag to the biosafety cabinet.				
3.03	**Test the filter with a diffusion test and a bubble point test according to the SOP. Add the results as an appendix**<br><br>Date and initials operator:				

4. Preparation of the B grade cleanroom

STEP	INSTRUCTIONS	REQ.	RESULT	INITIALS OP. 1	INITIALS OP. 2
4.01	**Collect the needed raw materials, disposables and utensils needed in the B grade cleanroom**				
	Transfer all needed materials to the B grade cleanroom.				
4.02	**Print four labels for step 5.08**				
4.03	**The steps below are performed in the B cleanroom**				
	Check if the cleanroom has been cleared for production				
	Report the clearance date in the room log.				
	Make sure the biosafety cabinet is turned on and cleaned according to the SOP.				
	Write down the serial number of the biosafety cabinet.				
	Write down the date the cabinet was cleaned.				
	Perform line clearance.				
	Date and initials:				

5. Aseptic filling of vials with IRDye 800CW solution

STEP	INSTRUCTIONS	REQ.	RESULT	INITIALS OP. 1	INITIALS OP. 2
5.01	**Preparing the biosafety cabinet for aseptic filling**				
	Place the Watson Marlow pump outside the biosafety cabinet and attach the Flexboy bag containing IRDye 800CW to a stand.<br><br>Place the materials and utensils in the biosafety cabinet in accordance with the image below.<br><br>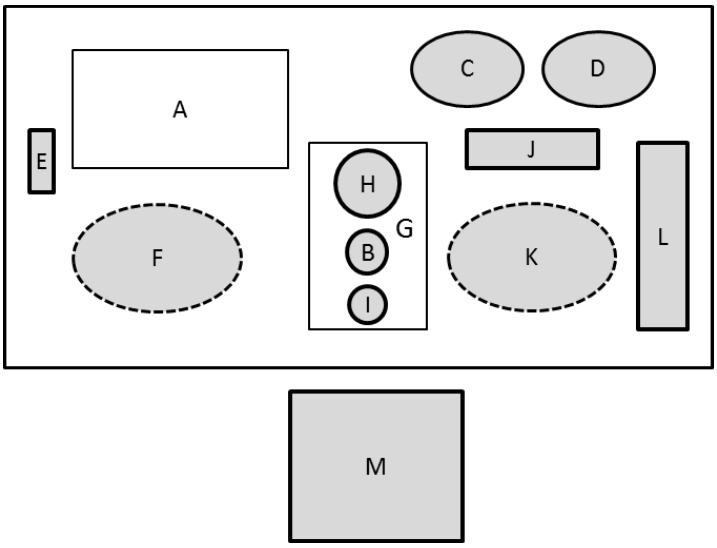<br><br>A. Tray with empty 10R vials<br>B. N/A<br>C. Bag containing sterile stoppers<br>D. Bag with sterile crimp caps<br>E. N/A<br>F. Working space for unpacking<br>M. Pump<br>G. Bulk container and filter<br>H. 800CW NHS ester solution<br>I. Millex GP filter (filling point)<br>J. Sterile pliers and AC/CP crimping tool<br>K. Working space for closing vials<br>L. Space for crimping tool / Cappress Jr.				
	Check the setup of the biosafety cabinet	Complies	Yes / No		

STEP	INSTRUCTIONS	REQ.	RESULT	INITIALS Op. 1	INITIALS Op. 2
5.02	**Check what hose set was last used in the pump logbook**				
	Is the pump already calibrated for the 1.6 mm tubing set? If so, proceed to step 5.03.	Pump calibrated for 1.6 mm?	Yes / No		
	If the pump was is calibrated for the 1.6 mm tubing set, calibrate the pump according to the SOP.<br>Calibration successful?	Calibration successful	Yes / No		
5.03	**Connect the tubing set for filling**				
	Connect the tube of the Flexboy bag with a Luer Lock connection to the tubing set for aseptic filling of 5 mL.<br>Insert the tubing set in the Watson Marlow pump.<br>Connect the Millex GP filter to the end of the tubing set. Make sure the filter is at the correct height for filling.				
5.04	**Flush the tubing set for priming**				
	Enter the following setting in the pump:<br>Speed: 100 rpm<br>Volume: 5.3 mL<br>Start ramp 3<br>End ramp: 0<br>Interval: 0; doses: 1<br>Tube size: 1.6 mm inner diameter	Settings correct?			

STEP	INSTRUCTIONS	REQ.	RESULT	INITIALS Op. 1	INITIALS Op. 2
	Take a 50 mL tube and insert it under the tip of the Millex GP filter.				
	Activate the pump using the pedal. Pump 2x 5,3 mL through the system to flush the system. Collect the product in a 50 mL tube. Close the tube after flushing and discard this.	2x 5,3 mL			
5.05	**Check the filling volume of the pump.**				
	Add all weighing report as an appendix.				
	Weigh 10 empty vials, stoppers and caps. Document the results				
	Weight 10 empty vials	Weight (a)	g		
	Weight 10 stoppers	Weight (b)	g		
	Weight 10 caps	Weight (c)	g		
	Calculate the average weight using the formula below				
	Weight empty vial (d) = (a + b + c)/10	Weight (d)	g		
	Calculate the lower and upper limit of the filling weight as shown below				
	Lower limit (g) = d + 5.3 g	Lower limit	g		
	Upper limit (g) = d + 5.7 g	Upper limit	g		
	Place a vial without defects at the filling point.				
	Activate the pump and fill the vial. Fill a total of 3 vials. Place a stopper and a crimp cap on the vials. Mark them as I, II and III				

STEP	INSTRUCTIONS	REQ.	RESULT	INITIALS OP. 1	INITIALS OP. 2
	Weigh the vials on the balance. Document the measured weights below.				
	Weight vial I		g		
	Weight vial II		g		
	Weight vial III		g		
	Calculate whether the weight of vials I – III falls within the acceptable limit. If the weight complies, begin the filling process and proceed to step 5.06. Document the average volume per vial.	5.3 – 5.7 mL	mL		
	If the weight of vials I-III is outside the acceptable limit, recalibrate the dosing pump according to the SOP. Refill three vials, cap them, and label them as IV, V, and VI. Weigh the vials on the balance				
	Weight vial IV		g		
	Weight vial V		g		
	Weight vial VI		g		
	Calculate whether the weight of vials IV – VI falls within the acceptable limits. If the weight complies, begin the filling process and proceed to step 2.06. If not, recalibrate the pump and until the weight of the vials complies and document the results in “Appendix B Notes”	5.3 – 5.7 mL	mL		
5.06	**Start filling ±120 vials**				
	**Person 1**: removes the vials from the package, places them under the GP Millex filter in the Biosafety Cabinet, and fills the vials using the Watson Marlow dosing pump and foot pedal	Initials person 1:			

STEP	INSTRUCTIONS	REQ.	RESULT	INITIALS Op. 1	INITIALS Op. 2
	**Person 2**: immediately after filling, use sterile tweezers to place a stopper on the vials and line the vials up. Seal the vials with a crimp cap after filling.	Initials person 2:			
	Replace the Millex GP filter with a new filter after every 50 filled vials. Mark the first vial filled after each filter change with an "A."				
	Repeat this process until the Flexboy bag containing the IRDye 800CW NHS ester solution is empty.				
	Mark the last vial filled with a "B."				
5.07	**Equip each filled vial with a rubber stopper and a crimp cap, and seal them using the semi-automatic crimping tool.**				
	Remove the vials marked with an “A” and vials I-VI from the biosafety cabinet and transfer them to a griploc bag.				
5.08	**Put the other capped vials back in the tray and remove them from the biosafety cabinet.**				
	Count the yield after filling and report the result				
	Remove the temporary labels from the vials with defects.				
	Mark the bottom of all the vials with a “1” using the Edding Permanent Marker 8280. Check the numbering **<u>IMMEDIATELY</u>** after marking using the UV-flashlight.				
	Also, record the corresponding defect number on the bottom of the respective vial, according to the numbering in Table 1 above. Check the numbering **<u>IMMEDIATELY</u>** after marking using the UV-flashlight.				
	Next, label the trays with vials and complete the label reconciliation table in Appendix A.				

STEP	INSTRUCTIONS	REQ.	RESULT	INITIALS Op. 1	INITIALS Op. 2
5.09	**Filter test**				
	Determine the bubble point of the used filters:<br>- Rinse each used filter with 10 mL of water<br>- Attach a syringe with 10 mL of air to the filter<br>- Submerge the tip of the filter under water<br>- Slowly depress the syringe and note the pressure at which air starts to escape from the filter				
	Bubble point filter 1	≤2.0 mL	mL		
	Bubble point filter 2	≤2.0 mL	mL		
	Bubble point filter 3	≤2.0 mL	mL		

APPENDIX A: LABEL RECONCILIATION

Example label:	Used label:
**QTS 800CW**<br>Date: ddMMMyyyy<br>Batch no: yyMdd-xxx<br>Store at 20 °C ± 5 °C<br>protected from light	

ACCOUNTABILITY OF LABEL QUANTITIES			
		**Total number produced:**	
**USED:**			
No. of labels on tray			
No. of labels on protocol			
No. of labels lost	N/A		
**TOTAL:**	=	**Total used:**	
		**Unused labels:**	+
		**Total**	=

	Datum	Sign
Operator:		

APPENDIX B: NOTES

Notes	Date	Initials
